# Occurrence of Multiple Psychiatric Comorbidities in a Child with Neurofibromatosis Type 1 (NF1): A Case Report

**DOI:** 10.7759/cureus.77745

**Published:** 2025-01-20

**Authors:** Afrah S Abedi, Nafisa Asad, Hejal Patel, Maleeha S Abedi, Ruhina Ali

**Affiliations:** 1 Psychiatry, Lake Erie College of Osteopathic Medicine, Bradenton, USA; 2 Internal Medicine, Lake Erie College of Osteopathic Medicine, Bradenton, USA; 3 Neurology, Lake Erie College of Osteopathic Medicine, Bradenton, USA; 4 Child and Adolescent Psychiatry, LifeStance Health, Naperville, USA

**Keywords:** adhd, autism spectrum disorder (asd), ddss, neurofibromatosis 1 (nf1), sensory stimulation

## Abstract

Neurofibromatosis type 1 (NF1) is a complex genetic disorder often associated with neurocutaneous manifestations and cognitive impairments. This case report examines a nine-year-old child with NF1 who presented with multiple psychiatric comorbidities, including autism spectrum disorder (ASD), attention-deficit/hyperactivity disorder (ADHD), and developmental disorder of scholastic skills (DDSS). The child exhibited significant impairments in daily functioning and academic performance. Comprehensive assessments identified deficits in social communication and repetitive behaviors consistent with ASD, as well as symptoms of inattention and hyperactivity indicative of ADHD. Furthermore, the child struggled with reading, writing, and mathematics, consistent with DDSS. This report highlights the importance of diagnostic evaluations in children with NF1 to identify and address co-occurring psychiatric conditions. The association between NF1 and these comorbidities suggests a similar neurobiological basis, potentially involving disrupted neural pathways and altered brain development. Early intervention strategies, including behavioral therapies, educational support, and appropriate pharmacological treatments, were implemented to address the child's needs. This case emphasizes the importance of personalized approaches to improve developmental, cognitive, and psychological outcomes for children with NF1 and multiple psychiatric comorbidities. Further research is necessary to better understand the mechanisms driving these associations and to guide treatment strategies.

## Introduction

Neurofibromatosis type 1 (NF1) is a common autosomal dominant genetic disorder, affecting approximately 1 in 3,000 individuals globally [1]. It results from mutations in the NF1 gene on chromosome 17, which encodes neurofibromin, a protein essential for regulating cell growth and differentiation [2]. While NF1 is primarily recognized for its dermatological and neurological manifestations, such as café-au-lait spots, neurofibromas, and Lisch nodules, its effects on cognitive and psychiatric health are becoming increasingly acknowledged.

Children with NF1 are at increased risk for various neurodevelopmental and psychiatric disorders, including autism spectrum disorder (ASD), attention-deficit/hyperactivity disorder (ADHD), and developmental disorders of scholastic skills (DDSS), such as dyslexia and dyscalculia [3]. ASD is characterized by impairments in social communication and interaction, along with restricted and repetitive behaviors. ADHD is marked by inattention, hyperactivity, and impulsivity, significantly affecting daily functioning and academic performance [4]. DDSS involve specific learning disabilities that impair a child's ability to read, write, or understand mathematics at age-appropriate levels. The occurrence of these psychiatric comorbidities in children with NF1 suggests overlapping etiological pathways [5]. Disruption of neurofibromin function may alter neuronal development and connectivity, contributing to a range of cognitive and behavioral anomalies. Early identification and management of these comorbidities are essential to mitigate their impact on the child's overall development and quality of life.

This case report describes a nine-year-old child with NF1 who presents with multiple psychiatric comorbidities, including ASD, ADHD, and DDSS. Through comprehensive clinical evaluation and intervention, this report highlights the importance of addressing the complex needs of children with NF1. The findings emphasize the need for strategies that target cognitive, behavioral, and educational challenges in these patients while encouraging future clinical practices and research directions.

## Case presentation

A nine-year-old male, diagnosed with NF1 at age six, presented with symptoms and developmental delays associated with the condition. He was born full term via cesarean section, weighing 7 lbs, and had a two-day hospital stay. In January 2023, he underwent open-heart surgery for atrial septal defect repair. His developmental milestones included sitting at six months, crawling at nine months, babbling at six months, speaking single words at three-and-half, achieving full speech at four years old, and being toilet-trained at three years old. 

The parents’ primary concerns focused on the patient's school behavior and performance, which are impacted by psychiatric comorbidities, including ASD, ADHD, and DDSS. When not on his prescribed medications (guanfacine 1 mg twice daily and dexmethylphenidate 5 mg daily), the patient exhibits behavioral issues such as difficulty keeping his hands to himself and using inappropriate language. His parents noted that after school, he becomes restless and experiences an increase in energy. His mother also reported a history of impulsivity, anger outbursts, difficulty sustaining attention, fidgeting, psychomotor agitation, forgetfulness in daily activities, distractibility, difficulty sitting still, excessive talking, interrupting others, loudness, difficulty organizing and completing tasks, and a lack of attention to detail. Caregivers observed increased distractibility but noted improvements in behavior during one-on-one interactions. The patient struggles with memory and retaining information, particularly with reading and problem-solving tasks related to mathematics. He adapts well to transitions and thrives when following a routine. Psychotherapy sessions focused on exploring the patient's mental health and well-being, discussing personal strengths, and developing strategies for managing stressors. The treatment plan also emphasized best practices to address symptoms and improve functional outcomes.

During the one-month follow-up, the patient's parents reported no improvements in his ADHD symptoms. Medication adherence has been minimal due to sensory sensitivities, including swallowing pills and heightened sensitivity to noxious stimuli. The patient had tried several medications, and his parents felt that methylphenidate XR 10 mg (4 mL of a 25 mg/5 ml liquid formulation) was the most effective, based on their observations and feedback from his teachers. However, teachers noted that the effects of methylphenidate XR 10 mg wore off by noon. As a result, methylphenidate IR 2.5 mg o(5 mg/5 mL oral solution) was added to his regimen, to be administered at lunchtime. 

Given the patient's history of developmental delay, a comprehensive occupational therapy (OT) evaluation was conducted, focusing on goals such as fine motor, visual, and sensory skills improvement, as well as correctly writing his name. Several assessments were performed as part of the evaluation to gain a comprehensive understanding of his condition. OT began by assessing the patient's reflexes, including supine flexion and prone extension. The patient was able to maintain the supine flexion position against gravity for approximately 12 seconds, while the age-appropriate norm is approximately 88 seconds, as shown in Table 1.

**Table 1:** Supine flexion normative and developmental guidelines Sec.: seconds, SD: standard deviation. Source: [6].

Age in years	Mean sec.	SD
4	10	14
5	25	20
6	46	27
7	68	37
8	88	54

The patient demonstrated difficulty maintaining the prone extension position, exhibiting knee and elbow flexion with significant rigidity, even when the therapist assisted. As a result, no score was recorded for their assessment. The average time for maintaining a prone extension position in this age group is 30 seconds, as shown in Table 2.

**Table 2:** Prone extension normative and developmental guidelines Sec.: seconds, SD: standard deviation. Source: [6].

Age in years	Mean sec.	SD
4	18.15	13
6	28.93	6
8	30	0

Despite the inability to gather data for a prone extension due to the aforementioned muscular compensations, the patient exhibits poor trunk flexor and extensor strength. Reflexes are automatic responses to sensory stimulation present in infants to facilitate development and are gradually integrated over time.

Furthermore, OT evaluated the patient’s functional impairments through activities of daily living (ADLs) and sensory profile results. The patient’s ADL performance is presented in Table 3, while the sensory profile results are displayed in Table 4. 

**Table 3:** Activities of daily living (ADLs) results

Area	Dependent	Needs assistance	Independent
Feeding	No	No	Yes
Grooming	No	No	Yes
Bathing	No	Yes	No
Upper extremity dressing	No	Yes	No
Lower extremity dressing	No	Yes	No
Toileting	No	No	Yes
Sleeping	No	Yes	No

**Table 4:** Sensory profile results

Area	Less than others	Just like others	More than others	Much more than others
Auditory processing	No	No	Yes	No
Visual processing	No	Yes	No	No
Tactile processing	No	Yes	No	No
Vestibular processing	No	No	Yes	No
Body position	No	Yes	No	No
Oral processing	No	No	No	Yes
Conduct	No	No	No	Yes
Social-emotional	No	No	No	Yes
Attentional	No	No	Yes	No
Seeking/seeker	No	No	Yes	No
Avoiding/avoider	No	No	No	Yes
Sensitivity/sensor	No	No	Yes	No
Registration/Bystander	No	No	Yes	No

Requiring assistance with ADLs significantly impacted the patient, exacerbating challenges related to executive function, motor coordination, and sensory processing. The patient's sensory profile, which indicates impairments "more than others" or "much more than others," heightens sensory sensitivities, leading to increased stress, anxiety, and difficulty managing daily environments. This hypersensitivity may interfere with the patient's ability to focus, regulate emotions, and engage socially, further complicating his ability to function independently. 

OT concluded that the patient's functional impairments affected his participation in play, work, and self-care. His primary functional impairments include sensory processing, executive functioning (attention, memory, and impulse control), fine motor skills, visual motor skills, ocular motor skills, body/safety awareness, and decreased upper body strength/endurance, all of which impact daily and school-related tasks. The causes of these functional impairments include weakness/muscular dysfunction, retained primitive reflexes, motor planning, ideation dysfunction, vestibular dysfunction, auditory dysfunction, poor bilateral integration, and executive function deficits. OT emphasized addressing these impairments through therapy could help improve the patient’s psychiatric comorbidities, particularly in areas of social interaction, self-care, and play skills. Therapy will target the limiting factors affecting the patient and work toward resolving each specific issue.

The therapy plan involves weekly sessions over 12 months, focusing on sensory processing, fine motor skills, emotional regulation, visual motor skills, play, and executive functioning. The primary goals include improving self-regulation, executive functioning, transitional skills, upper body strength, fine motor and visual motor skills, and the ability to complete age-appropriate tasks with minimal assistance and high accuracy.

## Discussion

NF1 is a genetic disorder characterized by neurocutaneous, skeletal, and cardiovascular abnormalities [2,3]. Emerging evidence indicates that NF1 is also associated with a range of neuropsychiatric comorbidities which present challenges to affected children and their families, highlighting the need for comprehensive management strategies. This case report examines a nine-year-old patient with NF1 who also exhibited comorbid conditions such as ASD, ADHD, and DDSS, highlighting the complex nature of NF1 and the need for early interventions.

The occurrence of ASD in children with NF1 has gained increasing recognition in recent years. Studies suggest that approximately 25% of children with NF1 meet the criteria for ASD, compared to around 1%-2% in the general population [7]. The overlap in symptoms, such as social communication deficits and restricted, repetitive behaviors, points to a shared neurobiological basis. NF1-related mutations disrupt the Ras/MAP kinase neural pathway, causing aberrant synaptic proteins and an imbalance between GABA and glutamate, which may lead to the observed ASD phenotypes [8]. ADHD is another common comorbidity in children with NF1, with prevalence rates reported as high as 50% [8,9]. While the exact mechanisms linking NF1 to ADHD are still under investigation, it is hypothesized that neurofibromin deficiency affects dopaminergic pathways and frontal lobe function, contributing to attentional deficits and hyperactivity [9]. DDSS, including dyslexia and dyscalculia, are also prevalent among children with NF1, affecting up to 60% of this population [10]. Research has linked increased T2 signal intensity on MRI, representing unidentified bright objects (UBO+), with developmental and learning deficits in patients with NF1. The presence of UBOs may indicate abnormal myelination and is associated with lower IQ, lower language scores, and impaired visuomotor integration and coordination in NF1 patients [11].

The relationship between NF1 and psychiatric comorbidities poses significant challenges for affected children and their families, profoundly impacting daily functioning and long-term outcomes. Children with NF1 and comorbid psychiatric disorders such as ASD, ADHD, and DDSS often experience developmental delays, cognitive impairments, and behavioral challenges that hinder social interactions, academic performance, and adaptive skills. These difficulties often exacerbate familial stress and caregiver burden, leading to increased emotional, physical, and financial strain within households [12]. Addressing these challenges requires a multidisciplinary approach that integrates educational, psychological, and medical interventions tailored to the specific needs of these children.

One promising intervention is diagnosis-specific school liaison programs (SLPs), which emphasize collaboration between clinical psychologists, school staff, and individualized educational plans (IEPs) [13]. Developed by the Multidisciplinary Neurofibromatosis Program at Boston Children’s Hospital, the Neurofibromatosis School Liaison Program (NF-SLP) aims to address educational gaps associated with NF1 [14]. The NF-SLP offers cost-free, consultative services to educate families and school teams about the educational and social challenges associated with NF1. It also assists in advocating for children’s educational rights and ensuring equitable access through accommodations and formal plans. An analysis of the first 200 individuals utilizing NF-SLP services highlights the program's efficacy. Following NF-SLP input, 100% of patients received expanded educational services or accommodations, and 98% now have formalized plans such as IEPs or 504 Plans, 31 of which were newly created. General education services are supplemented by 504 Plans, which include classroom accommodations, while IEPs provide direct interventions from special education teams. These measures address common academic challenges faced by children with NF1, such as difficulties with executive functioning, attention, and social cognition. However, further research is necessary to assess the long-term effectiveness of these programs and compare outcomes across different intervention strategies. 

Studies have demonstrated improvements in patients with concurrent NF1 and attentional issues through pharmacological management. A randomized controlled trial comparing methylphenidate to placebo showed short-term benefits in NF1 children with academic challenges and attention deficit [15]. This effect could potentially be enhanced with specific cognitive training targeting attention. Lidzba et al. tested the hypothesis that long-term methylphenidate treatment could improve cognitive functioning in children with NF1 and co-occurring ADHD [16]. The study retrospectively analyzed patients with NF1, with or without ADHD, who underwent standardized neuropsychological assessments twice. Using repeated measures analysis of covariance (rmANCOVA), the study found that medicated children and adolescents with NF1 showed significant cognitive improvement, while those without ADHD did not exhibit notable changes. Furthermore, Houpt et al. highlighted the limited data on optimal treatment strategies for existing psychiatric comorbidities in NF1 [4]. This study aimed to address that gap in understanding and exploring the neurobiological basis of psychiatric comorbidities in NF1. The use of second-generation antipsychotics or hydroxyzine was associated with more frequent behavioral health-related emergency department visits, admissions, and inpatient days. On the other hand, stimulant medications, particularly methylphenidate, significantly improved cognitive and academic performance, as well as social deficits, while reducing ADHD symptoms in children and adolescents with NF1.

In addition to pharmacological interventions, physical, occupational, and speech therapies play a crucial role in addressing the gross and fine motor delays often observed in patients with NF1 [17]. Common motor challenges include difficulties with coordination, balance, muscle tone, and handwriting skills, all of which can significantly impact daily functioning and academic performance. Early initiation of these therapies, in collaboration with other healthcare providers, is essential for improving developmental outcomes and mitigating the long-term effects of NF1-related neurodevelopmental challenges.

A review by Farahani highlights the efficacy of psychosocial therapy programs in improving the quality of life (QoL) for children and adolescents with NF1, particularly in areas where they experience the most profound deficits compared to the general population [18]. Programs such as the Relaxation Response and Resiliency Program (3RP), Creative Arts Therapy, and Acceptance and Commitment Therapy (ACT) have been shown to address multiple domains of QoL, as assessed by the SF-36 questionnaire, which includes physical functioning, social functioning, vitality, bodily pain, general health perception, and mental health. Among these therapies, the 3RP stands out for improving all six domains, while ACT and Creative Arts Therapy demonstrate significant benefits in addressing bodily pain and mental health. Given that depression and anxiety account for 32% of the variance in QoL within the NF1 population, focusing on mental health through these complementary therapies is particularly crucial.

Although significant progress has been made in treating patients with NF1, further research and development are needed to continue improving the QoL and develop new therapeutic programs tailored to the individual needs of patients. It is essential to prioritize sustained intervention efforts throughout childhood and adolescence to longitudinally support developmental needs and maximize outcomes of patients with NF1. Exploring the integration of these therapies into standard care protocols, their scalability, and their cost-effectiveness will be essential for maximizing their accessibility. Investigating how these approaches can be utilized to meet the diverse needs of NF1 patients and their families will further contribute to the development of comprehensive care models.

## Conclusions

In conclusion, the association between NF1 and multiple psychiatric comorbidities, including ASD, ADHD, and DDSS, emphasizes the need for a comprehensive management approach. Early identification and intervention are necessary to improve the developmental and educational outcomes of children with NF1. Multidisciplinary teams play a pivotal role in addressing the needs of these patients, varying from educational support, improvements at home, and access to various therapies. Further research is required to gain a better grasp of the neurobiological mechanisms underlying these associations and to develop respective treatment plans. Understanding the full spectrum of NF1-related comorbidities will allow support for affected individuals and their families.
